# Model structure–activity relationship studies of potential tropane 5HT_1A_, 5HT_2A_, and D_2_ receptor ligands

**DOI:** 10.1007/s00044-012-0305-6

**Published:** 2012-11-11

**Authors:** Tomasz Słowiński, Jacek Stefanowicz, Martyna Z. Wróbel, Franciszek Herold, Andrzej Mazurek, Franciszek Pluciński, Aleksander P. Mazurek, Irena Wolska

**Affiliations:** 1 Department of Drug Technology and Pharmaceutical Biotechnology, Medical University of Warsaw, 1 Banacha Str., 02-097 Warsaw, Poland; 2 National Medicines Institute, 30/34 Chełmska Str., 00-725 Warsaw, Poland; 3 Department of Drug Chemistry, Medical University of Warsaw, 1 Banacha Str., 02-097 Warsaw, Poland; 4 Department of Crystallography, Faculty of Chemistry, Adam Mickiewicz University, 6 Grunwaldzka Str., 60-780 Poznan, Poland

**Keywords:** D_2_ receptor, Tropane derivatives, Antipsychotics, SAR

## Abstract

The two-stages studies of structure–activity relationship for model ligands of 5HT_1A_, 5HT_2A_, and D_2_ receptors were performed. On the first stage, the pharmacophores of two potential ligands of known in vitro binding to 5HT_1A_, 5HT_2A_, D_2_ receptors and model pharmacophore of strongly interacting D_2_ receptor ligands were found and their parameters were related to affinity data. The analyzed parameters were hydrophobic, hydrophilic, aromatic, donor and acceptor of proton centers. The geometry of spatial distribution of these properties was also investigated in comparative analysis. The studied, model compounds were two 3β-acylamine derivatives of tropane. The second stage includes docking of studied compounds to D_2_ receptor model and the comparison of its quality with in vivo binding data. The obtained results are consistent with in vitro binding data and applied procedure accurate estimates the affinity of potential ligands to D_2_ receptors.

## Introduction

**Fig. 1:**
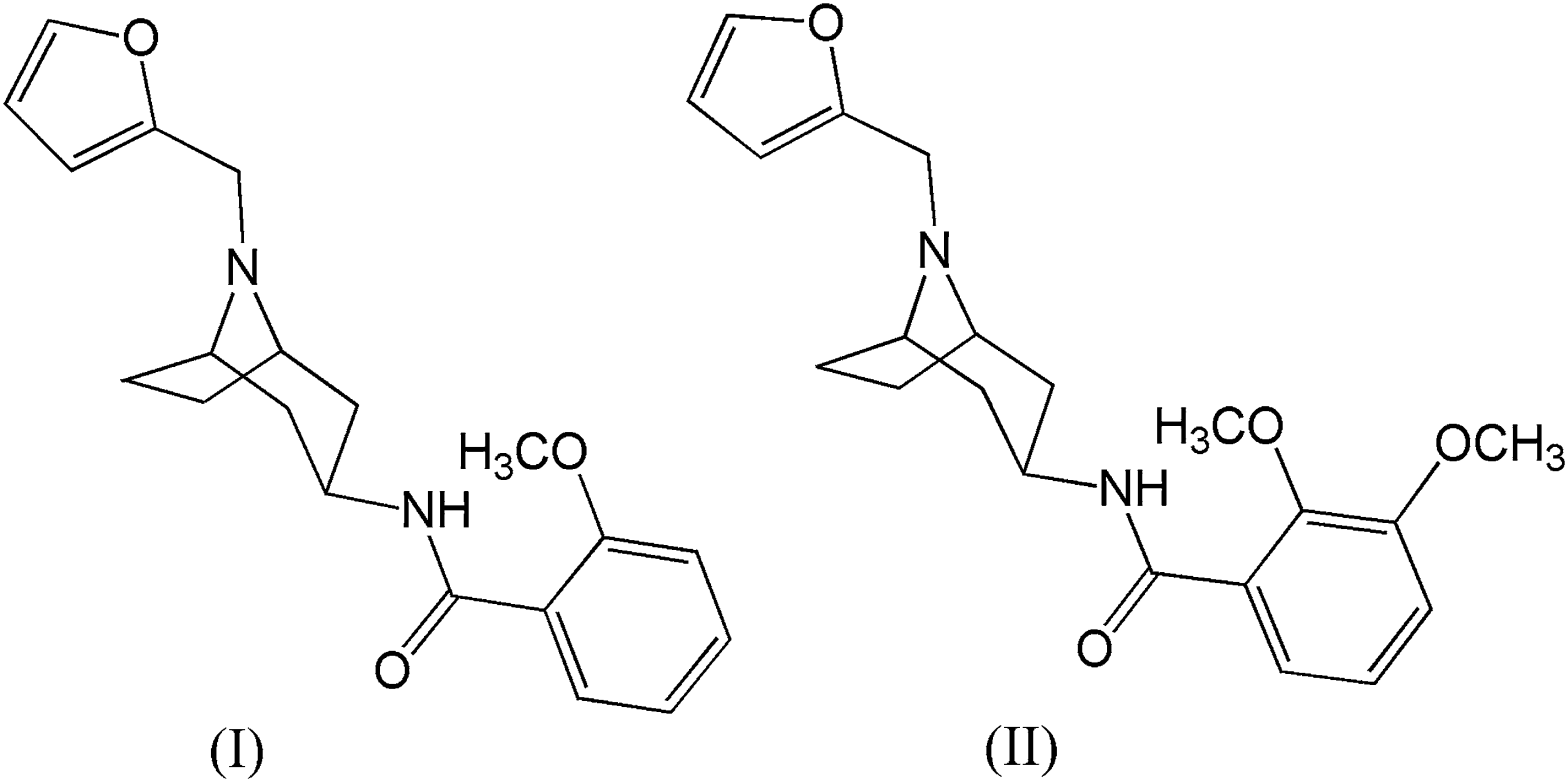
The chemical formulas of compound **I** and compound **II**

In commonly accepted opinion every searching for new, more effective drugs should be rationalized i.e., determined by the low cost and non time-consuming procedures. These procedures are especially useful on the preliminary stage of searching for new chemical structures of potential biological activity (Jorgensen, 2004; Leeson and Springthorpe, 2007; Ou-Yang *et al.*, 2012). In general, on this purpose there are employed various correlation QSAR methods (Dudek *et al.*, 2006; Yang and Huang, 2006; Shailesh *et al.*, 2012). However, in particular cases it is more convenient to develop the procedure of selection of the appropriate structures based on more direct and easier interpretatively criteria. It seems that just such a case is a search for effective ligands of 5HT_1A_, 5HT_2A_, and D_2_ receptors since many structural data on their agonist and antagonist as well as the models of these receptors are well-known (Klabunde and Hessler, 2002; Bissantz *et al.*, 2003; Teeter *et al.*, 1994; Chambers and Nichols, 2002; Homan *et al.*, 1999). In addition, wide availability of various bases containing a lot of structural data on very active ligands allows to generate pretty accurate pharmacophore patterns (Nelson, 1991; Bojarski, 2006). Thanks to these all literature data it is possible to estimate the affinity of potential ligand for receptor of interest. The chemical structure of pharmacophore of being selected potential ligand and its affinity to the receptor seem to be sufficiently unambiguous discriminators, on a preliminary stage, in the search for new effective antipsychotics. To verify this hypothesis, the two-step procedure was developed and tested. The first step includes determination of pharmacophores for two tested compounds of well-known affinity (previously in vitro determined) to the same receptors as well as pharmacophore pertinent to well-known D_2_ receptor agonists or antagonists and finally comparison of their properties to in vitro binding data. The pharmacophore model of D_2_ receptor ligands was found on the basis of 15 compounds of high affinity to D_2_ receptor reported in literature (Słowiński *et al.*, 2011). These two tested compounds were 3β-acylamine derivatives of tropane: *N*-(8-Furan-2-ylmethyl-8-azabicyclo[3.2.1]oct-3β-yl)-2-methoxybenzamide (compounds **I**) and *N*-(8-Furan-2-ylmethyl-8-azabicyclo[3.2.1]oct-3β-yl)-2.3-dimethoxybenzamide (compound **II**) (Fig. 1). Their synthesis have been developed and described in the previously published paper on tropane derivatives (Słowiński *et al.*, 2011).

**Fig. 2:**
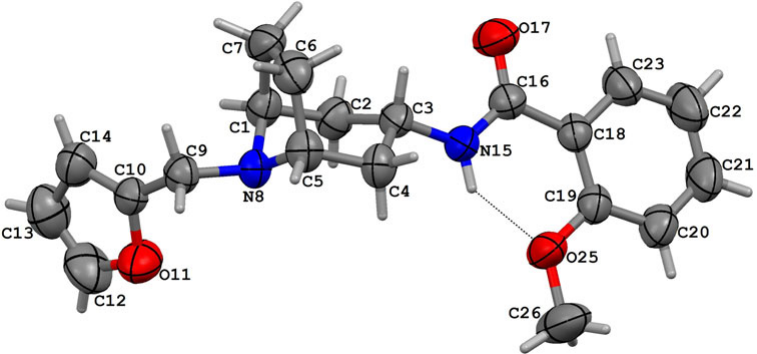
The X-ray diffraction structure of compound **I**

**Fig. 3:**
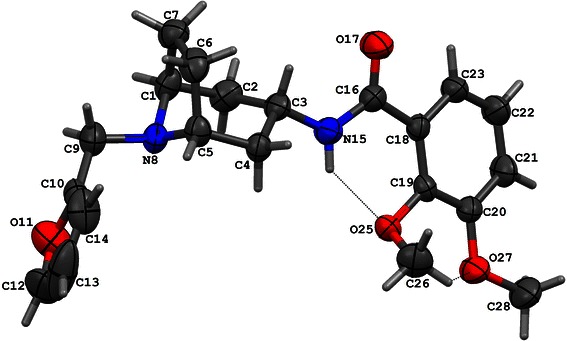
The X-ray diffraction structure of compound **II**

The pharmacophores of compounds **I** and **II** were found on the basis of their structures determined by X-ray diffraction method. The CCDC (Cambridge Crystallographic Data Centre) numbers of compounds **I** and **II** are: 905689 and 905690, respectively (Figs. 2, 3).

The molecular structure of compound **I** shows an intramolecular hydrogen bond between the O atom of the methoxy group and the NH of the amide function leads to a six-membered ring. The dihedral angle between the least-squares planes of the phenyl and this virtual ring is only 2.50(7)°. The piperidine moiety adopts a chair conformation. The substituent at N8 is in an equatorial position. The best plane of the furan ring and the C1/C2/C4/C5 plane make an angle 69.42(9)° and the dihedral angle between the planes of the furan and benzene rings is 72.50(8)°.

The compound **II** molecule adopts a folded conformation with an angle between the furan and benzene rings of 63.29(8)° and between the best plane of the furan ring and the C1/C2/C4/C5 plane of 87.56(9)°. This conformation is stabilized by an intramolecular N15–H15A···O25 and C26–H26C···O27 hydrogen bonds. As a result of N15–H15A···O25 interaction a six-membered ring is formed and make an angle 9.2(1)° with the phenyl ring. The piperidine moiety assumes a chair conformation and the substituent at N8 is in an equatorial position. Conformations of both methoxy groups are different. The disposition of these groups with respect to the phenyl ring can be described by the torsion angles C18–C19–O25–C26 of −107.8(2)° and C21–C20–O27–C28 of 11.1(3)°. In consequence, the methyl carbon atom C26 is found to be 1.107(4) Å out of the phenyl plane, and C28 atom is almost coplanar with this ring.

The pharmacophore structure is a reflection template of the geometrical distribution of property centers localized in molecule and determines to large extent its biological activity. It means that even subtle differences in the geometry of structurally similar molecules can significantly impact on their affinity to receptor binding site.

The comparative analysis of the studied pharmacophores was intended to find the specific properties and geometrical parameters which are crucial for the strength of binding of potential ligands to the receptors of interest.

The second step of the applied procedure devoted to the selection of the potential agonists or antagonists of the studied receptors relies on docking of the reference compounds **I** and **II** to the models of the D_2_ receptor (Sakhteman *et al.*, 2011). From analysis of in vitro results (Table 1) follows that the both studied compounds (**I**, **II**) are very poorly being bounded to 5-HT_1A_ and 5-HT_2A_ receptors. Indeed, the model docking of compounds **I** and **II** to these receptors also showed that such binding cannot take place. The both molecules of compounds **I** and **II** were placed outside the receptor binding pockets. Thus, only docking of compounds **I** and **II** to D_2_ receptor is detailed analyzed. The most discriminative parameters which distinctly classify the quality of docking are number and strength (equivalently length and geometry) of the hydrogen bonds formed between ligand and specific amino acids not only inside the receptor binding pocket but also, although to a less degree, intermolecular interactions of other types e.g., hydrophobic and edge-to-face.

**Table 1:** 5HT_1A_, 5HT_2A_, and D_2_ receptor affinities

Ligand	Receptor [K(nM)]		
	5HT_1A_	5HT_2A_	D_2_
Compound **I**	6,100	6,000	1,000
Compound **II**	3,000	744.5	26.3

The used 3D homology model of D_2_ receptor has been revealed by comparative modeling using the crystal structure of the human β_2_-adrenergic receptor and the bovine rhodopsin as the templates (Sakhteman *et al.*, 2011; Strzelczyk *et al.*, 2004; Wang *et al.*, 2010). The quite recently reported X-ray structure of the human β_2_-adrenergic receptor opens new possibilities for modeling of the correct structures of the dopamine ones. Currently, the human β_2_-adrenergic receptor is considered to be more homologous to the dopamine receptors than bovine rhodopsin (Cherezov *et al.*, 2007). All modeling of the pharmacophores as well as docking of the compounds **I** and **II** to the D_2_ receptor model were done by Discovery Studio software (Accelrys Software Inc., Discovery Studio Modeling Environment, 2005).

## Materials and methods

### X-ray diffraction measurements

Crystals of compounds **I** and **II** suitable for X-ray analysis were grown by slow evaporation from acetate/diisopropyl ether (compound **I**) and hexane/ethanol (compound **II**) solutions. The data were collected on an Oxford Diffraction KM4CCD diffractometer at 293 K, using graphite-monochromated Mo K_α_ radiation. The unit cell parameters were determined by least-squares treatment of setting angles of highest-intensity reflections chosen from the whole experiment. Intensity data were corrected for the Lorentz and polarization effects. The structure was solved by direct methods using the SHELXS97 program (Sheldric, 1990) and refined by the full-matrix least-squares method with the SHELXL97 program (Sheldric, 1997). The function Σ*w*(|*F*_o_|^2 ^− |*F*_c_|^2^)^2^ was minimized with *w* ^−1 ^= [σ^2^(*F*_o_)^2^ + (0.0688*P*)^2^], where *P* = (*F*_o_^2^ + 2*F*_c_^2^)/3. An empirical extinction correction was also applied according to the formula *F*_c_′ = k*F*_c_[1 + (0.001χ*F*_c_^2^λ^3^/sin2θ)]^−1/4^ (Sheldric, 1997) and the extinction coefficient χ was equal to 0.014(2). All non-hydrogen atoms were refined anisotropically. The coordinates of the hydrogen atoms were calculated in idealized positions and refined as a riding model with their thermal parameters calculated as 1.2 (1.5 for methyl group) times U_eq_ of the respective carrier carbon atom.

## Results and discussion

The in vitro binding data for compounds **I**, **II** as ligands of 5HT_1A_, 5HT_2A_, and D_2_ receptors are given in Table 1 (Słowiński *et al.*, 2011).

**Fig. 4:**
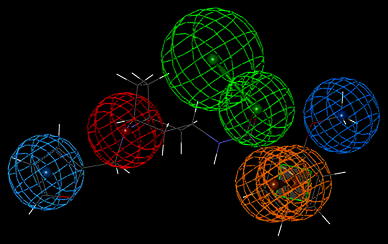
The spatial distribution of pharmacophore properties on a background of compound **I** X-ray diffraction structure. A *green square* depicts the plane of a phenyl ring (Color figure online)

**Fig. 5:**
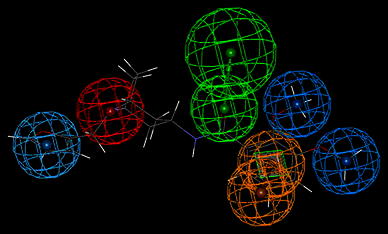
The spatial distribution of pharmacophore properties on a background of compound **II** X-ray diffraction structure. A *green square* depicts the plane of a phenyl ring (Color figure online)

**Fig. 6:**
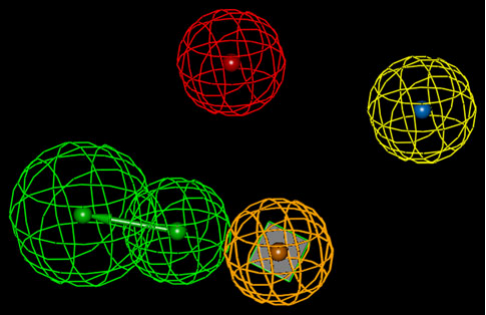
The spatial distribution of pharmacophore properties of D_2_ receptor ligands. A *green square* depicts the plane of a phenyl ring. The *yellow sphere* stands for hydrophobic—aliphatic property (Color figure online)

These experimental binding data unambiguously points at very low affinity of compound **I** to 5HT_1A_ and 5HT_2A_ receptors and somewhat better to D_2_ one, yet, compound **II** displayed very weak binding activity to 5HT_1A_, moderate to 5HT_2A_ and very high to D_2_ receptors. The differences between parameters (geometrical and property types) of the reference pharmacophores and the pharmacophores pertinent to compounds **I** and **II** are expected to reflect the differences in affinity of tested compounds to the receptors of interest. The found structures of pharmacophores described by their specific properties are given on—Figs. 4, 5, and 6. The particular colors denote the following properties: red—positive ionization (nitrogen atom), green—hydrogen bond acceptor, magenta—hydrogen bond donor, pale blue—hydrophobic, aromatic, ultramarine—hydrophobic, aliphatic, orange—aromatic ring (Table 2).

**Table 2:** Pharmacophore properties of compound **I** and **II**

Pharmacophore feature/property	Compound **I**	Compound **II**
Positive ionization (red)	Nitrogen atom	Nitrogen atom
Hydrogen bond acceptor (HBA, green)	Carbonyl group of amide bond	Carbonyl group of amide bond
Aromatic ring (orange)	Benzene ring substituted with methoxy group	Benzene ring substituted with two methoxy groups
Hydrophobic, aromatic (pale blue)	Furane ring	Furane ring
Hydrophobic, aliphatic (ultramarine)	One methyl group in methoxy moiety attached to the benzene ring	Two methyl groups in methoxy moieties attached to the benzene ring

**Fig. 7:**
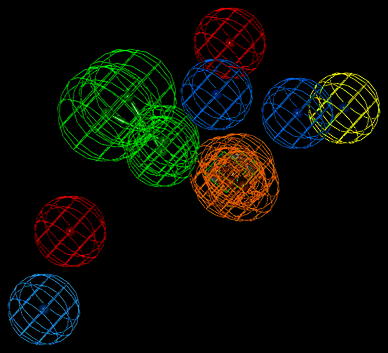
Superposition of the D_2_ receptor ligand pharmacophore and pharmacophore of compound **II**

The geometry of a spatial distribution of pharmacophore properties in obtained models is an exact reflection of the X-ray diffraction structure of compounds **I** and **II** (Table 3). It is worthy to note that in spite of the high similarity of chemical structures of these compounds, that their conformations significantly differ each from other. Consequently, these differences distinctly appear in pharmacophore models. Obviously, it should be taken into account some flexibility of the spatial pharmacophore geometry and possibility of its change during docking of studied compounds to particular receptors. However, such changes are often possible only to small degree or impossible at all on account of the high energetic rotation barriers. In this context, the presence of two separate aliphatic—hydrophobic centers in pharmacophore of compound **II** takes on a special importance for explanation of very high affinity of this compound, in contrast to compound **I**, for D_2_ receptor. It is likely that just second methoxy group in compound **II** molecule underlies its high binding to D_2_ receptor while the same group do not affect the affinity of compound **II** to 5-HT_1A_ and 5-HT_2A_ receptors. The comparative analysis of the D_2_ receptor ligand pharmacophore (Fig. 6) and pharmacophores of compounds **I** and **II** also leads to the same conclusion (Figs. 4 and 5). The pharmacophore of D_2_ ligand quite well matches the pharmacophore of compound **II** but does not the pharmacophore of compound **I** (c.f. Fig. 7). In addition, specificity of the structural relation between these pharmacophores results from the identical spatial localization of the aliphatic property of D_2_ ligand pharmacophore and its analog present in pharmacophore of compound **II** but absent in pharmacophore of compound **I** (Table 2).

**Table 3:** Pharmacophore geometry parameters

Pharmacophore geometry parameters	Compound **I**	Compound **II**
Distance between piperidine nitrogen atom and center of the benzene ring	7.85 Å	7.76 Å
Dihedral angle between benzene ring plane and furane ring plane	72.50°	63.29°
Dihedral angle between piperidine ring (C1/C2/C4/C5) plane and benzene ring plane	65.79°	50.97°
Dihedral angle between piperidine ring (C1/C2/C4/C5) plane and furane ring plane	69.42°	87.56°
Dihedral angle between carbonyl group plane and piperidine ring plane	73.50°	86.72°

**Fig. 8:**
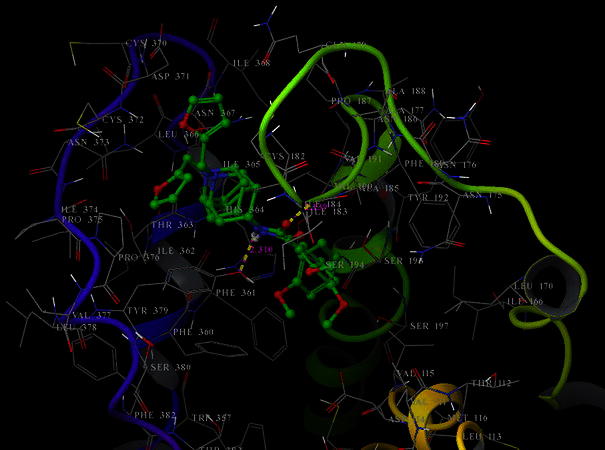
The molecules of compounds **I** and **II** (*green*) inside binding pocket of D_2_ receptor. *Yellow dashed lines* denote hydrogen bonds (Color figure online)

Docking of both tested compounds to D_2_ receptor model turned out to be non discriminative investigation not giving criteria for explanation of difference in ability to the binding of compounds **I** and **II** with D_2_ receptor. Both compounds docked to D_2_ receptor interact with its amino acids via the same hydrogen bonds. In case of compound **I** the hydrogen bonds are: ligand—thyrosine 379 (length 2.198 Å), ligand—alanine 185 (length 2.315 Å), and compound **II** ligand—thyrosine 379 (length 2.310 Å), ligand—alanine 185 (length 2.139 Å). In addition, both compounds interact similarly with D_2_ receptor with hydrophobic forces (Fig. 8).

The obtained docking results are not unexpected since, purposely, the structurally similar compounds were investigated to point out that even very subtle differences in the chemical structure of compounds, to which docking procedure is “insensitive”, may impact crucially on their therapeutic activity. Thus, it should be stated that two stages “pharmacophore” and “docking” investigations are necessary to estimate properly an affinity of newly designed receptor ligands. On the whole, these studies were intended to prove that postulated two-stages procedure can be applied to verification of the properties of even very similar structurally potential and being designed antipsychotics.
